# Psychometric properties of a nationwide survey for adults with and without diabetes: the “disease knowledge and information needs – diabetes mellitus (2017)” survey

**DOI:** 10.1186/s12889-020-8296-6

**Published:** 2020-02-06

**Authors:** Lena M. Stühmann, Rebecca Paprott, Christin Heidemann, Thomas Ziese, Sylvia Hansen, Daniela Zahn, Christa Scheidt-Nave, Paul Gellert

**Affiliations:** 1 Department of Epidemiology and Health Monitoring, Robert Koch Institute, Berlin, Germany; 2 Charité – Universitätsmedizin Berlin, Institute for Medical Sociology and Rehabilitation Science, Berlin, Germany; 3 Ceres - Cologne Center for Ethics, Rights, Economics, and Social Sciences of Health, Cologne, Germany; 4 Federal Centre for Health Education (BZgA), Office for National Education and Communication on Diabetes Mellitus, Cologne, Germany; 5 Preventive Cardiology and Medical Prevention, Department of Cardiology, University Medical Centre, Johannes Gutenberg University Mainz, Mainz, Germany

**Keywords:** Diabetes mellitus, Health monitoring, Population-based survey, Psychosocial, Health care, Germany

## Abstract

**Background:**

In order to close existing information gaps on diabetes-related health perceptions, diabetes knowledge, and information-seeking behaviors among adults in Germany, a representative population-based survey targeting the German-speaking population 18 years and older with and without diabetes was conducted. The aim of the present work was to analyze the psychometric properties of the multi-item scales, applied in the survey in order to provide guidance for decisions on the use of these measurements for future research.

**Methods:**

Based on data from participants who completed the final survey (*N* = 1479 with known diabetes; *N* = 2327 without known diabetes) reliability and unidimensionality of multi-item scales were tested using Cronbach’s Alpha and confirmatory factor analysis (CFA).

**Results:**

Psychometric properties and model fit varied across scales. Cronbach’s alpha values ranged from very good to unacceptable. Model fit indices suggested evidence of a single underlying factor in some but not all scales. Adequate reliability and at least mediocre model fit were found for diabetes distress and patient-provider-relationship in people with diabetes and for perceived level of information in individuals without diabetes. Scales revealing inacceptable reliability values or not suggesting unidimensionality were e.g. diabetes-related stigmatization in both individuals with and without diabetes, self-efficacy in individuals with diabetes, and perceived personal control in those without diabetes.

**Conclusion:**

Based on results of the current study, some of the scales applied in the survey can be recommended for present and future analyses of the survey data and for future surveys (e.g. diabetes distress, patient-provider-relationship in people with diabetes). Other scales should be interpreted and used with caution (e.g. depressive symptoms in people with diabetes) while others should be reformulated, interpreted only as single items, or need further investigation (e.g. diabetes-related stigmatization in people with and without diabetes). Findings provide researchers the opportunity to evaluate diabetes-specific scales in population-based studies of adults with and without diabetes.

## Background

Diabetes mellitus, in particular type 2 diabetes is a chronic metabolic disease of great Public Health impact in Germany and worldwide [1, 2]. The disease requires lifelong treatment and self-management challenging both the affected individuals and the society [3].

Extensive health actions are required on a national level to halt the rise in diabetes and its related consequences such as complications, comorbidities and premature mortality [4, 5]. Diabetes monitoring with population-based surveys on a national level is important to inform national diabetes policies and public health strategies focusing on the prevention of diabetes and related secondary health problems [4]. Previous research has shown that there is growing evidence on the impact of psychosocial and health care factors on diabetes-self management among adults with diabetes [5–7]. Likewise, in people without diabetes, psychosocial factors and health care-related factors, such as physician counselling or participation in diabetes prevention programs appear to be associated with diabetes preventive behaviors and outcomes, e.g. physical activity or weight loss [8–13].

There is a lack of population-based surveys, which assess a broad range of diabetes-related concepts that include not just clinical factors related with diabetes, but include psychosocial and environmental factors as conceptualized in the International Classification of Functioning, Disability and Health (ICF) model [14, 15]. This model is a valuable framework for structuring our understanding of factors associated with a specific condition including physiological and psychological functioning, activities and participation in social life [15], which, in turn, are related to personal and environmental factors, representing an individuals’ whole life background. These factors comprise sociodemographic factors, lifestyle, habits, past and current experience and other characteristics, but also social structures, health service use, and social attitudes. A small number of national and international population-based surveys of individuals with diabetes exist that focus on psychosocial and health care factors that represent the personal and environmental level [16–23]. However, few surveys so far have focused on specific diabetes-related information needs and information seeking behavior in people with and without diagnosed diabetes. (For a review of categories from leading international diabetes surveys, see Additional file 1).

In order to fill the gap of research on information needs and information seeking behavior in the context of psychosocial and environmental factors, the German “Disease Knowledge and Information Needs – Diabetes mellitus (2017)” survey was conducted among individuals with and without diabetes. This survey was part of the national diabetes surveillance system initiative [24]. First results of the survey are presented elsewhere [24].

To gain a broad understanding of potential interrelations between diabetes-related information needs, information seeking behavior, disease knowledge and other relevant health-related concepts a set of other personal and environmental factors, e.g. health beliefs, risk perceptions, health care utilization or patient-provider-relationship was included in the survey by drawing on the ICF model [14, 15], and reviewing existing surveys and expert discussions. While instruments to tap these concepts were available from the literature in many albeit not all cases, there was a general lack of information on psychometric properties in population-based settings. However, such knowledge is the key to inform and support survey-based in-depth analyses on information needs and disease knowledge, but also on several of the concepts measured by multi-item measurements as outcome variables. Moreover, the results will also be used to guide future use of these instruments in subsequent population-based surveys of diabetes-related knowledge and information needs in Germany and internationally.

## Methods

### Study design and sample

The survey “Disease knowledge and information needs – Diabetes mellitus (2017)”, was conducted from August to December 2017 by the Robert Koch Institute (RKI) in cooperation with the Office for National Education and Communication on Diabetes Mellitus of the Federal Centre for Health Education (BZgA), and the Institute of Medical Sociology and Rehabilitation Science of the Charité – Universitätsmedizin Berlin. This project was funded by the Federal Ministry of Health as part of a larger project to establish a national diabetes surveillance system and improved risk communication to the public [25]. The survey was conducted as a health telephone survey by applying two standardized interviews, i.e. one for people without known diabetes and one for people with self-reported physician diagnosed diabetes. The target population consisted of German residents who were at least 18 years old and had sufficient German language skills to participate in a telephone interview. For the survey, the intended sample size was *N* = 1500 for individuals with diabetes and *N* = 2500 for individuals without diabetes. The sampling procedure was realized in two phases using an established dual-frame methodology considering landline as well as mobile telephone numbers in order to obtain representativeness for all potentially reachable private households at a national level. In a first main survey phase, a sample of the general adult population was drawn, i.e. including people with and without diabetes. The Kish Selection Grid method was applied, i.e. target persons were randomly selected from multi-person households by a computer-assisted algorithm that was based on the number of adults in a household and the sequence of their age. This procedure ensured the same selection probability for all potential target persons. Respondents were assigned to one of the two survey components, i.e. people with and without diagnosed diabetes, based on the question “Have you ever been diagnosed with diabetes by a physician?” (“yes” or “no”). More detailed information on the sampling procedure can be found elsewhere [24, 26]. This sampling phase resulted in complete interviews of 263 individuals with diabetes and 2327 individuals without diabetes. The response rate calculated according to the American Association for Public Opinion Research (AAPOR), i.e. the proportion of conducted interviews related to all potentially reachable households in Germany, either via landline or mobile telephone, was 17.9% [24].

In the second main survey phase a sample of only individuals with diabetes was drawn by applying a direct screening procedure, i.e. people were asked whether they or another person in the household had ever been diagnosed with diabetes. In case of more than one person with diabetes per household, the Kish Selection Grid method was used as well. A total of 1479 interviews of people with diabetes was conducted. Overall, the final survey sample comprised 2327 individuals without known diabetes and 1479 individuals with a history of diagnosed diabetes.

Data were collected from August to November of 2017 through computer assisted telephone interviews (CATI) by the market and social research institute USUMA GmbH in Berlin, Germany. Interviews were performed by trained interviewers. Participation in the survey was voluntary. Individuals received information on survey procedure and data protection. After that, they were asked to give verbally informed consent about their willingness to participate.

### Measures

For both survey groups, i.e. one comprising individuals with known diabetes and the other one comprising individuals without known diabetes, a customized questionnaire was applied to each group. In order to tap identified concepts and constructs of the survey, validated and short German language items and instruments were chosen, if available. If selected constructs were not represented adequately by already existing instruments or only by extensive scales or were not available in German language, items were newly developed or adapted from existing ones by the research team or were translated from existing English language instruments following a forward-backward procedure [27]. Moreover, survey development included cognitive testing of selected items to test comprehensibility and acceptance. Following the results of the testing, e.g. a “do not know” category was added for some items because respondents noted, they would choose the middle category due to that missing answer option. Final revisions of items were conducted following an initial data collection phase based on difficulties that interviewers noted when conducting the interviews. Final versions of interviews took on average 32 min to complete for individuals without diabetes and 43 min for individuals with diabetes. A complete list of concepts, constructs, and instruments as applied in the main survey can be found elsewhere [24].

The present study focused only on multi-item measures and scales that were used in the main survey. Among people with diabetes these were: the optimistic bias subscale of the Risk Perception Survey-Diabetes Mellitus (RPS-DM) [28]; the personal control subscale of the Revised Illness Perception Questionnaire (IPQ-R) [29, 30]; the self-care ability subscale which was adopted from the Diabetes Care Profile (DCP) [31]; diabetes-related stigmatization with two items adopted from the Diabetes Representative Survey [32] and a new item based on the Type 2 Diabetes Stigma Assessment Scale (DSAS-2) [33]; the Problem Areas in Diabetes Scale – Five-item Short Form (PAID-5) [34]; the Two-item Patient Health Questionnaire (PHQ-2) [35]; the Patient Assessment of Chronic Illness Care-DAWN Short Form (PACIC-DSF) [36]; and the Information Needs in Diabetes Questionnaire (IND) [37]. Among people without diabetes these were: the optimistic bias subscale of the Risk Perception Survey-Developing Diabetes (RPS-DD) [38]; the personal control subscale of the RPS-DD [38]; diabetes-related stigmatization with two items adopted from the Diabetes Representative Survey and a new item based on the DSAS-2 [33]; actual diabetes knowledge with two items adopted from Hoghton et al. [39] and four new items; and the IND [37] (with only 5 items).

### Statistical analyses

Descriptive statistics were reported for sample characteristics and all scales under study. Descriptive statistics of items not incorporated into scales are reported elsewhere [24]. Psychometric properties were determined for the scales. All analyses were performed based on data weighted with sample weights in order to achieve representativeness at the national level as described in detail previously [24]. Individuals who reported a diagnosis of diabetes by a physician at some point previously in life, but no presence of diabetes within the last 12 months and no current medication as well as individuals with a current gestational diabetes were excluded from the analyses (*n* = 83).

Means and standard deviations were reported for the scales. Reliability was indicated using Cronbach’s alpha coefficient for scales with more than two items. Values of Cronbach’s alpha lower than .60 were interpreted to be unacceptable, between .60 and .65 were considered undesirable, between .65 and .70 minimally acceptable, between .70 and .80 respectable, and values between .80 and .90 were considered very good based on suggestions for research instruments [40]. For scales comprising two items, the Spearman-Brown coefficient equivalent to the standardized coefficient alpha was calculated [41]. The Spearman-Brown coefficient was calculated for the optimistic bias subscale of the RPS-DM [28] and the PHQ-2 [35] in individuals with diabetes, and for the optimistic bias subscale of the RPS-DD in individuals without diabetes. Analyses were done using the statistic software SPSS (IBM SPSS v.22.0). Additionally, the unidimensional factorial structure of the scales used in the survey components was tested by applying confirmatory factor analysis (CFA), since Cronbach’s Alpha is not recommended to examine unidimensionality [42, 43]. Therefore, the packages “lavaan” and “lavaan.survey” in R v3.4.3 were used. First, lavaan.survey was utilized to incorporate the sampling weights. Then, to test unidimensionality factor scales were modeled as single-factor models. For parameter estimation, the robust maximum likelihood estimator “MLM” was chosen to take the non-normality of the data into account. The model fit was evaluated by considering an absolute fit index, the robust root-mean-square error of the approximation (RMSEA) and an incremental fit index, the robust comparative-fit-index (CFI) [44] since they belong to the commonly used fit indices [45–47]. RMSEA values under .05 [34] or .06 [28] indicate good model fit, according to widely used rules of thumb [45, 48, 49]. Accordingly, values between .05 and .10 [32] or .05 and .10 [34] indicate mediocre model fit while values above .10 indicate poor model fit [34]. For the CFI, rules of thumb suggest good model fit occurs with values above 0.95 [44, 50] or, preferably, 0.97 [50]. CFA analyses were done on scales comprising at least four items, as a minimum of four indicators measuring one latent factor is necessary for a single-factor model to be overidentified [51]. For scales comprising less than four items only reliability coefficients were calculated as described before.

Missing data from scale variables were examined and treated separately for the two subgroups of individuals with and without diabetes. Missing values were less than 5% for most items. Items with more than 5% of the values missing included both items of the RPS-DM optimistic bias subscale (8.0 and 11.7%) in individuals with diabetes as well as one item each of the RPS-DD optimistic bias subscale (7.2%) and the RPS-DD personal control subscale (5.6%) in individuals without diabetes. Missing data were assumed to be missing at random. Missing data were estimated by applying the expectation-maximization algorithm within SPSS, using age and sex as predictors as well as the weight variable and all scale variables of the respective subgroup. This method suited the complex data structure, including filter variables, by using a single data set when applying missing data treatment, survey weights analyses for CFA using lavaan.survey, and estimations of Cronbach’s alpha coefficient. The expectation-maximization algorithm has shown superiority over case deletion methods [52].

## Results

### Study population

In the sample of 1396 individuals with diabetes, the proportion of women and men was comparable (49.9% vs. 50.1%) (Table 1). Participants had a mean age of 65.3 years (SD = 13.8). About one in two was married and lived with their partner (50.2%). Almost half of the sample (46.8%) had a low educational level while 13.5% of the participants were highly educated. In this sample, 79.2% of the participants stated they had type 2 diabetes, 14.0% had type 1 diabetes, and a few reported another type of diabetes (1.3%), while 5.5% reported they did not know. Average diabetes duration was 14.7 years (SD = 11.0). More than half of the participants reported to have none of the complications related to diabetes presented in the survey (58.4%) whereas 34.7% reported at least one complication. The mean BMI was 29.4 kg/m^2^ (SD = 5.5). In the sample of *N* = 2327 individuals without diabetes the proportion of women and men was similar as well (51.7% vs. 48.3%). The mean age was 49.6 years (SD = 18.6). While 30.7% of the participants had a low educational level, 26.9% were classified as highly educated. Participants in this sample had a mean BMI of 25.5 kg/m^2^ (SD = 4.4).

**Table 1:** Sample characteristics for individuals of the parallel survey components

	People with diabetes (*n*^a^ = 1396)	People without diabetes (*n*^a^ = 2327)
Age in years, mean (SD)	65.3 (13.8)	49.6 (18.6)
Sex (% female)	49.9	51.7
Relationship status		
Married, living together with partner	50.2	46.2
Married, living apart	2.4	3.0
Single	13.1	32.5
Divorced/separated	10.5	9.1
Widowed/partner died	23.7	8.9
Educational level^b^		
Low	46.8	30.7
Average/medium	39.6	42.2
High	13.5	26.9
Migration background		
No migration background	88.4	80.3
Onesided (one parent was born in another country than Germany)	4.8	4.8
Twosided (Both parents or individual itself plus one parent were born in another country than Germany)	6.6	14.7
Living area		
< 20.000 citizen	43.6	41.5
≥ 20.000 citizen	43.0	47.2
Diabetes type (self-reported)		
Type 1	14.0	
Type 2	79.2	
Others	1.3	
Diabetes duration (years), mean (SD)	14.7 (11.0)	
Current treatment^c^		
Glucose-lowering tablets	61.5	
Insulin	50.2	
Other glucose-lowering medicine being injected	4.9	
Diet/healthy nutrition	58.0	
Physical activity/sports	52.9	
No treatment	0.6	
Diabetes related complications		
No complication	58.4	
One or more of the requested complications^d^	34.7	
Other complications than requested	2.4	
Comorbidities^e^		
No comorbidities	57.7	
One or more comorbidities	41.1	
BMI in kg/m^2,^ mean (SD)	30.2 (6.0)	25.6 (4.6)

*Note*. Data are given as weighted and are reported in percent if not stated otherwise. Missing values based on unweighted data were ≤ 10% for living area in individuals with and without diabetes. Missing values were ≤ 5% for all other variables

^a^Sample sizes are reported unweighted

^b^Comparative Analysis of Social Mobility in Industrial Nations (CASMIN) classification was used to report educational level

^c^Multiple answers were possible

^d^Complications asked in this survey were kidney disease, eye disease, nervous disease, diabetic foot lesions and amputations

^e^Comorbidities asked in this survey were heart attack, stroke, coronary heart disease, depression diagnosed by a physician/psychotherapist

### Scale distributions

Distributions and ranges of scales from both survey components are summarized in Additional file 2. In individuals with diabetes, scale means approximated the theoretical scale mean (i.e., on a 1–2–3-4-5 scale the theoretical mean would be 3) for: relative risk perception (i.e., optimistic bias) of getting complications (M = 2.6; SD = 2.7), patient-provider-relationship in terms of the patients’ assessment of care (M = 2.5; SD = 1.0), and the perceived level of information (M = 2.7; SD = 0.6). Scale means were below the theoretical scale mean for diabetes-related stigmatization (M = 2.0; SD = 0.8), diabetes distress (M = 3.4; SD = 4.3), and depressive symptoms (M = 1.2; SD = 1.6). Scale means of perceived personal control (M = 16.0; SD = 2.8) and self-efficacy (M = 3.6; SD = 0.4) were higher than the theoretical scale mean. Additional scale information on diabetes-related stigmatization is given in Additional file 3: Table S1.

In those without diabetes, the scale mean of perceived personal control (M = 1.9; SD = 0.5) was below the theoretical scale mean. For relative risk perception (i.e., optimistic bias) of developing diabetes (M = 2.3; SD = 0.7), diabetes-related stigmatization (M = 2.5; SD = 0.7), actual diabetes knowledge (M = 2.5; SD = 1.6) and perceived level of information (M = 2.3; SD = 0.7) scale means were close to or only slightly below their theoretical scale means. Additional scale information on diabetes-related stigmatization and actual diabetes knowledge are given in Additional file 3: Tables S2 and S3.

### Reliability and factorial structure

A complete overview of the psychometric properties of the survey measures for people with and without diabetes is given in Additional file 2. For measures applied among persons with diabetes, scale reliability as assessed by Cronbach’s alpha values ranged from .53 for self-efficacy to .90 for perceived level of information. Values for perceived level of information (α = .90), diabetes distress (α = .88), patient-provider-relationship in terms of the patients’ assessment of care (α = .85), perceived personal control (α = .76), and relative risk perception of getting complications (α = .71) were within the range from respectable to very good reliability. For self-efficacy, depressive symptoms, diabetes-related stigmatization, and health literacy Cronbach’s alpha values ranged from unacceptable to minimally acceptable. The lowest value returned for diabetes-related stigmatization (α = .52).

Using CFA to further test the unidimensional factorial structure of scales in participants with known diabetes, RMSEA values ranging from .06 for self-efficacy to .21 for perceived personal control were found. The CFI value was lowest for perceived level of information (CFI = .90) and highest for self-efficacy (CFI = .98). The model test for perceived personal control revealed an RMSEA = .21 and a CFI = .91, both indicating poor model fit. The same applies to perceived level of information (RMSEA = .11 and CFI = .90). For diabetes distress, the RMSEA was .13, which indicates poor fit, whereas the CFI was .97 which indicates good fit. The model fit indexes for patient-provider-relationship in terms of the patients’ assessment of care (RMSEA = .07 and CFI = .96) and self-efficacy (RMSEA = .06 and CFI = .98) indicated mediocre to good fit.

For individuals without diabetes, values of scale reliability ranged from .31 to .91. Cronbach’s alpha reliability coefficients of the relative risk perception of getting diabetes (α = .65) and perceived level of information (α = .91) can be described as minimally acceptable and very good, respectively. Perceived personal control, diabetes-related stigmatization, and actual diabetes knowledge returned unacceptable to undesirable Cronbach’s alpha coefficients, with diabetes-related stigmatization having the lowest value (α = .31).

Applying CFA for the assessment of unidimensionalty in the sample of individuals without diabetes, the fit index RMSEA ranged from .07 for perceived level of information to .19 for perceived personal control. Likewise, the CFI ranged from .73 for perceived personal control to .99 for perceived level of information. For perceived personal control, the resulting RMSEA of .19 and CFI of .73 indicated poor fit. Regarding actual diabetes knowledge, an RMSEA of .10 and CFI of .94 similarly indicated poor fit. For perceived level of information, the RMSEA of .07 indicated mediocre fit while the CFI of .99 indicated good fit.

In the present study, we tested unidimensional scales. Although beyond of the scope of the study, examples of alternative scale structures for specific scales can be found in Additional file 4: Tables S1 and S2.

## Discussion

The present study investigated the psychometric properties for multiple item measures used in a survey that focused on disease knowledge and information needs as well as related factors as conceptualized in the ICF model [14, 15]. Scales were administered in individuals with diabetes, in individuals without diabetes, or in both groups depending on scale content. Based on the evaluation of reliability coefficients and factorial structures, which were found in this study, we provide recommendations for the use of the investigated multiple item measures or scales in both people with and without diabetes in future national or international population-based surveys on diabetes prevention and care.

### Evaluation of scales by means of reliability in conjunction with factorial structure

In participants with diabetes, for some scales respectable-to-very good reliability coefficients but also poor or mixed justification for a single underlying dimension were found. This may have been occurred for several reasons. For example, for the perceived personal control subscale of the German IPQ-R whose reliability was largely in line with the results of the evaluation of the German IPQ-R [30], poor values of fit indices of the CFA might have been associated with length of the subscale and associated low number of degrees of freedom [53, 54].

For the PAID-5, reliability was in line with findings by McGuire et al. [34], whereas mixed results of model fit did not fully support the one-factor solution of an exploratory factor analysis found by McGuire et al. [34] or the one-factor model found through the application of CFA in a Korean study [55]. However, different results may be explained by model modification that was applied in the Korean study but not in the current study or by different administration modes of the scale. In the Korean study, the PAID-5 was administered to participants in written form whilst in the current study it was administered verbally over the phone [55]. Thus, the mode of administration as well as further analyses of model fit including model modifications should be addressed in future research.

For the perceived level of information, lack of evidence for unidimensionality may be explained by the fact that the primary aim of the IND is to identify information gaps in individuals with diabetes and changes in their information needs over time. Overall, in the current study the factor-analytical results suggest single items to be used, instead of a composite score. Nonetheless, results indicated unidimensionalty for perceived level of information in individuals without diabetes, which was assessed with a reduced number of items of the IND. Thus, there might be potential to generate a score that assesses an overall perceived level of information on diabetes.

Other scales in people with diabetes showed moderate or good model fit but differed in their reliability. For diabetes-specific self-efficacy, which was assessed with the modified self-care ability scale [31], reliability appeared to be low whereas Fitzgerald et al. [31] found respectable reliability in a US sample. Items may have been less interrelated in the current study due to the modification of a single item or translation processes. However, the low scale reliability should be taken into account when applying or interpreting this scale. Another scale, the PACIC-DSF, however, showed good reliability.

In people without diabetes, in contrast to the perceived level of information, the personal control subscale and actual diabetes knowledge seemed to perform poorly in terms of reliability as well as model fit. The personal control subscale showed a lower Cronbach’s alpha coefficient than the one found for a US sample with an English version and for a Spanish sample with a Spanish version of the RPS-DD [56]. Moreover, Joiner et al. [57] found the personal control scale to be unidimensional only after removing two of the four items, when applying exploratory principal components analyses. Translation of the scale into German language or the conception of items among the German population may have caused additional heterogeneousness. However, disparity among items could also indicate that alternative factorial structures fit the data more adequately and should be further investigated when applied to the German population.

For actual diabetes knowledge, results suggest that a single latent factor model representing a single dimension of diabetes knowledge may not optimally fit the data. A model taking into account different facets of the items assessing diabetes knowledge such as knowledge related to biological mechanisms of diabetes, knowledge specific to type 2 diabetes, and specific to type 1 diabetes might be more suitable to fit the data as people might have knowledge regarding one diabetes type but not the other. The use of specific knowledge questions (i.e., single items) for specific types of diabetes may be recommended.

### Evaluation of scales based on reliability only

Relative risk perception was assessed in people with and without diabetes, using group-specific optimistic bias scales. Reliability coefficients found in the current study were in line with results of a prior study using an English version of the optimistic bias scale in people without diabetes [38] on the one hand. One the other hand they were slightly lower or lower than in studies applying an English or Spanish version in people with diabetes [28] and without diabetes [57]. For our survey, items of the optimistic bias subscales have been translated into German language. Although the rate of missing data for items of both optimistic bias scales was acceptable for a telephone survey, it was higher than in any other items of scales investigated in this study. Missingness of the optimistic bias items is in part likely due to the complexity of phrasing and thus missingness response patterns should carefully be inspected if the optimistic bias subscales will be applied in a future telephone survey.

The PHQ-2 was used to assess depressive symptoms in individuals with diabetes. In this instrument, one item represented the lowering of mood and another the lack of interest. Results of the present study indicated lower reliability than the results found in a primary care sample by Löwe et al. [58]. Items seemed to be more heterogeneous in the sample of individuals with diabetes compared to the primary care sample, which included individuals who did not necessarily have diabetes. As Furuya et al. [59] suggested, two-item questionnaires assessing depressive symptoms could show different characteristics among different populations such as in individuals with or without diabetes and thus should be further investigated. Based on our findings, we cannot give an unconditional recommendation for the use of the two-item depression scale among those with diabetes.

For diabetes-related stigmatization in individuals with and without diabetes unacceptable reliabilities and unclear factorial structures, as these could not be tested due to the insufficient number of items, suggest that items may not be used to generate a scale score representing overall stigmatization but may be used as single item measures. In future studies, items could be complemented by additional items and psychometric properties should be tested again. Otherwise, already existing longer scales assessing stigmatization might be an alternative [33].

### Recommendations for scale use

A broad range of reliability values of the survey’s scales was found. Similarly, when investigating the unidimensional factorial structure of scales using CFA, a broad range of model fits were found, suggesting evidence of a single underlying factor in some but not all scales. According to the results of this study, we distinguished several classes of scales in terms of psychometric properties. The first class showed very good reliability and at least mediocre model fit, indicating that these scales provide sound results and can be recommended for the application in further analyses and surveys. This class includes scales of diabetes distress (i.e. the PAID-5) and patient-provider-relationship in terms of the patients’ assessment of care (i.e. the PACIC-DSF) in the survey component for individuals with diabetes. The perceived level of information scale administered in individuals without diabetes may also be allocated to the first class, based on its psychometric properties, but needs further exploration. Overall, the good reliability and the mean structure allow us to carefully recommend the PACIC-DSF to be used for repeated measurements. Similarly, the IND [37] may be used to monitor change in overall perceived levels of information in case that future research efforts will produce a scale applicable to assess the overall level of information as explained before. However, future research should address the responsiveness of these scales.

For a second class of scales, results produced by the application of these scales should be interpreted with caution. Moreover, psychometric properties might need to be further investigated in other samples and pretests before including scales of this class into a survey. In individuals with diabetes, scales in this class include scales for relative risk perception (i.e. optimistic bias), perceived personal control, and depressive symptoms. In individuals without diabetes, scales in this class include relative risk perception (i.e. optimistic bias).

For a third class of scales, items should probably not be interpreted as a scale score but should rather be interpreted as single items. Alternatively, scales should be modified or extended by adding further items or scales could be replaced by more reliable but possibly longer already existing scales. For individuals with diabetes, this concerns self-efficacy, diabetes-related stigmatization, and perceived level of information. For individuals without diabetes this refers to perceived personal control, actual diabetes knowledge, and diabetes-related stigmatization.

### Strength and limitations

Strengths of the survey include that it was conducted on a national level following a highly standardized survey recruitment protocol and weighting procedures of the RKI, allowing for the conclusions to be representative for the German population. Another strength of the present study is the use of Cronbach’s alpha in conjunction with CFA in order to evaluate the psychometrics properties of the multiple item measures. Interpretation of Cronbach’s alpha is more reasonable when the underlying structure of a scale is known, since Cronbach’s alpha does not measure dimensionality [42, 60, 61]. Moreover, Cronbach’s alpha can be used to confirm unidimensionality after the same has been suggested by factorial analyses [61, 62].

Several limitations need to be considered. Extended survey length represents a burden for participants and possibly influences the quality of their answers [63]. Therefore, valid instruments often could not be included in full length but only as subscales or single items. This partly resulted in lower reliabilities, as seen in the diabetes-related stigmatization scale in individuals with and without diabetes and in single factor models with only a few indicators. Because there were less than four indicators per factor, unidimensionality could not be tested for some scales. In other scales including only four or five indicators per factor, model fit indices might have been influenced by the small number of degrees of freedom, resulting in higher rejection rates of the model. Including several subscales belonging to the same instrument in a factorial model might help to identify models that fit the data adequately. Further, scales examined in this study were assumed to be unidimensional and thus tested by specifying models with a single latent factor. However, models incorporating multiple factors or underlying restrictions might be more suitable to fit the data.

Beyond unidimensional solutions, future studies should further investigate the possibility of multi-dimensional solutions. For instance, for actual diabetes knowledge, a three-factor-solution revealed better model fit compared to the single-factor-solution with knowledge related to biological mechanisms of diabetes, knowledge specific to type 2 diabetes, and knowledge specific to type 1 diabetes were distinct subscales. For the perceived personal control scale, alternative model structures, e.g. including a method effect, were conceivable as well, as this scale comprised two items phrased positively and two items phrased negatively. Another alternative model might comprise two factors with two indicators each. Of those factors one might represent a perceived controllability of risk of getting diabetes based on one’s own efforts. The other one might represent the perception of health or diabetes risks as uncontrollable inescapable fate independent of one’s own efforts. Hence, researchers using scales from this survey without excellent fit indicating unidimensionality are recommended to further explore the structure of these scales.

## Conclusions

Taken together, a range of psychometric properties across scales was found based on data of population-based survey of diabetes-related knowledge and information needs for both people with and without known diabetes. Some scales have shown adequate reliability and unidimensionality and are therefore recommended for future repeated survey waves. Other scales should be used and interpreted with caution, while a few scales should be reformulated or used as single item measures. Findings of the survey might serve to monitor diabetes-related factors at the population level, to select valid instruments that allow for incorporating the patient perspective into health surveillance systems, and to provide future researchers the opportunity to evaluate diabetes-specific scales among population-based samples of adults with and without diabetes.

## Supplementary information

**Additional file 1.** Comparison between concepts/constructs of population-based diabetes surveys. The table comprises an overview of large national and international population-based surveys on diabetes which were compared in terms of the assessed concepts and constructs.

**Additional file 2.** Scale distributions, reliability and unidimensional factor analyses for scales used in both survey components. The table presents scale distributions, Cronbach’s Alpha coefficient as well as results of confirmatory, i.e. unidimensional factor analyses for scales used in the two survey components: people with diagnosed diabetes and people without diagnosed diabetes.

**Additional file 3: Table S1, S2 and S3.** For questions on disease-related stigmatization in both people with and without known diabetes and actual diabetes knowledge in people without known diabetes, numbers of participants that selected “don’t know” as a response or refused to answer as well as corresponding sample characterization are presented in **Table S1, S2 and S3**.

**Additional file 4: Table S1 and S2.** Exemplary post-hoc structural model modifications were applied to actual diabetes knowledge and perceived personal control which in people without known diabetes.

## Data Availability

The authors confirm that some access restrictions apply to the analytical data set underlying our current findings. First, informed consent from the survey participants did not cover public deposition of data. Second, publicly providing an anonymized version of the analytical data set used in our current analysis would not comply with current data protection regulations in Germany, as anonymized information could still be used in combination to identify survey participants. Thus, the analytical data set underlying the findings is archived in the Research Data Centre at the Robert Koch Institute (RKI) and can only be accessed on-site by interested researchers at the Secure Data Center of the RKI’s Research Data Centre. Requests should be submitted to the RKI Research Data Centre, Robert Koch Institute, Berlin, Germany (e-mail: fdz@rki.de).

## Abbreviations

BMI: Body Mass Index
BZgA: Bundeszentrale für gesundheitliche Aufklärung (German Federal Centre for Health Education)
CATI: Computer Assisted Telephone Interview
CFA: Confirmatory Factor Analysis
CFI: Comparative Fit Index
DAWN2: Diabetes Attitudes Wishes and Needs 2
DCP: Diabetes Care Profile
IND: Information Needs in Diabetes Questionnaire
IPQ-R: Revised Illness Perception Questionnaire
PACIC-DSF: Patient Assessment of Chronic Illness Care-DAWN Short Form
PAID-5: Problem Areas in Diabetes Scale - Five-item Short Form
PHQ-2: Two-item Patient Health Questionnaire
RMSEA: Root Mean Square Error of Approximation
RPS-DD: Risk Perception Survey-Developing Diabetes
RPS-DM: Risk Perception Survey-Diabetes Mellitus
SPSS: Statistic Package for the Social Science
