# Supplementary material for: Psychometric properties of a nationwide survey for adults with and without diabetes: the “disease knowledge and information needs – diabetes mellitus (2017)” survey
Source: BMC Public Health. 2020 Feb 6;20:192. doi: 10.1186/s12889-020-8296-6 (PMC7006078; doi:10.1186/s12889-020-8296-6)
Supplement: Supplementary file 1 — Additional file 1. Comparison between concepts/constructs of population-based diabetes surveys. The table comprises an overview of large national and international population-based surveys on diabetes which were compared in terms of the assessed concepts and constructs. [file 12889_2020_8296_MOESM1_ESM.docx]

Additional File1

| **Table**  *Comparison between concepts/constructs of population-based diabetes surveys.* | | | | | | | | | | |
| --- | --- | --- | --- | --- | --- | --- | --- | --- | --- | --- |
| Concepts/constructs | Diabetes surveys | | | | | | | | | |
|  | **ABCD [1]** | **DAWN2 [2]** | **MILES – Australia [3]/ MILES-2 Survey [4]^a^** | **DISTANCE [5]** | **FDS [6]** | | **DIABASIS [7]** | **LWDS [8]** | **SLCDC-D [9]** | **Disease knowledge and information needs – diabetes^b^** |
| Country | Alberta (Canada) | 17 countries | Australia | California (United States) | Western Australia | | France | Queensland (Australia) | Canada | Germany |
| Sample size (N) | 2040 | 9040 | 3338/2342 | 20188 | 1732 | | 1092 (diabetes)  12943 (no diabetes) | 3951 | 2335 | 1396 (diabetes)  2327 (no diabetes) |
| Target group | Type 2 | Type 1 and type 2 | Type 1 and type 2 in both | only gestational diabetes excluded | No type specified | | Type 2 and general population | Type 1 and type 2 | Type 2 | All types and general population |
| Inclusion age in years | ≥18 | ≥18 | 18-70/18-75 | 30-75 | Not specified | | ≥45 | ≥18 | ≥20 | ≥18 |
| Administration mode | Online question-naire | online questionnaire, telephone or face-to-face interview | online or postal questionnaire in both | telephone interview, online or written questionnaire | telephone interview, postal questionnaire | | mailed self-report questionnaire | mailed self-report questionnaire | telephone interview | telephone interview |
| **Individual disease characteristics** | | | | | | | | | | |
| Clinical diabetes profile | ✓ | ✓ | ✓^c,d^ | ✓ | ✓ | ✓ | | ✓ | ✓ | ✓ |
| Membership of diabetes organization |  |  | ✓^c,d^ |  |  |  | |  |  |  |
| **Diabetes self-management** | | | | | | | | | | |
| Self-Care behaviour | ✓ | ✓ | ✓^c,d^ | ✓ |  | ✓ | | ✓ |  | ✓ |
| Detailed information on blood glucose monitoring |  |  | ✓^c^ | ✓ |  |  | |  |  | ✓ |
| Conducive or unconducive aspects for self-management | ✓ | ✓ | ✓^c,d^ |  |  |  | | ✓ |  |  |
| **Disease perceptions and believes** | | | | | | | | | | |
| Risk perception |  |  |  |  |  |  | |  |  | ✓ |
| Beliefs |  | ✓ | ✓^c^ | ✓ |  | ✓ | |  |  | ✓ |
| Diabetes-specific self-efficacy | ✓ | ✓ | ✓^c,d^ |  |  |  | |  |  | ✓ |
| Diabetes-related perceived stigmatization |  | ✓ | ✓^d^ |  |  |  | |  |  | ✓ |
| **Knowledge about disease** | | | | | | | | | | |
| Perceived diabetes knowledge |  |  |  |  |  |  | |  |  | ✓ |
| Actual diabetes knowledge |  |  |  | ✓ | ✓ | ✓^e^ | |  |  |  |
| Knowledge about diabetes due to occupation |  |  |  |  |  |  | |  |  | ✓ |
| **Health-related behavior** | | | | | | | | | | |
| BMI (weight, height) | ✓ | ✓ | ✓^c,d^ | ✓ |  | ✓ | | ✓ |  | ✓ |
| Behavioral factors (e.g., activity, diet, smoking) | ✓ |  | ✓^c,d^ | ✓ | ✓ | ✓ | | ✓ | ✓ | ✓ |
| Vaccination status | ✓ |  |  |  |  |  | |  |  |  |
| Health app-use |  |  | ✓^d^ |  |  |  | |  |  | ✓ |
| **Psychological aspects and health** | | | | | | | | | | |
| Reaction following diagnosis |  |  |  |  |  | ✓ | |  |  |  |
| Diabetes burden | ✓ | ✓ | ✓^c,d^ |  | ✓ | ✓ | | ✓ |  | ✓ |
| Illness centrality |  |  | ✓^d^ |  |  |  | |  |  |  |
| Quality of life/health status^f^ | ✓ | ✓ | ✓^c,d^ | ✓ | ✓ | ✓ | | ✓ |  | ✓ |
| Well-being |  | ✓ | ✓^1,2^ |  |  |  | |  |  |  |
| Pain | ✓ | ✓ |  | ✓ |  |  | |  |  |  |
| Mental health (e.g., depressive symptoms) | ✓ | ✓ | ✓^c,d^ | ✓ | ✓ |  | | ✓ |  | ✓ |
| Personality and Self (e.g., self-esteem) |  |  | ✓^c,d^ | ✓ |  |  | | ✓ |  |  |
| **Social environment** |  |  |  |  |  |  | |  |  |  |
| Social support |  | ✓ | ✓^c,d^ | ✓ |  | ✓ | | ✓ |  | ✓ |
| Partner with diagnosed diabetes |  |  |  |  |  |  | |  |  | ✓ |
| **Health care** | | | | | | | | | | |
| Availability and access to care | ✓ |  | ✓^c^ | ✓ | ✓ |  | | ✓ |  |  |
| Healthcare/physician utilization | ✓ | ✓ | ✓^c,d^ | ✓ | ✓ | ✓ | | ✓ |  |  |
| Sources of health care | ✓ | ✓ | ✓^c,d^ |  |  | ✓ | |  |  |  |
| Clinical monitoring by health care professional | ✓ | ✓ |  | ✓ |  | ✓ | | ✓ | ✓ | ✓ |
| Patient-provider-relationship | ✓ | ✓ |  | ✓ |  | ✓ | | ✓ |  | ✓ |
| Providing recommendations on health behaviors by a physician |  | ✓ |  | ✓ |  |  | |  | ✓ | ✓ |
| Needs for technological access (e.g. video consultation) |  |  | ✓^c^ |  |  |  | |  |  |  |
| Awareness of and attendance at disease management program (DMP) |  |  |  |  |  |  | |  |  | ✓ |
| Attendance at training/education program |  | ✓ | ✓^c,d^ |  |  |  | |  |  | ✓ |
| Diabetes-related costs | ✓ | ✓ | ✓^c^ | ✓ |  |  | | ✓ |  |  |
| **Diabetes-related information** | | | | | | | | | | |
| Diabetes related health information |  | ✓ |  |  |  | ✓ | |  |  | ✓ |
| Health literacy/aspects of health literacy | ✓ |  | ✓^c^ | ✓ |  |  | |  |  | ✓ |
| *Note***.** Similar constructs were subsumed under more general categories if applicable. The table does not claim to be complete in terms of existing population-based surveys regarding diabetes.  **ABCD, the Alberta’s Caring for Diabetes Study**; **DAWN2, Diabetes Attitudes Wishes and Needs 2: MILES-Australia, the Diabetes Management and Impact for Long-term Empowerment and Success – Australia 2011 SurveyMILES-2, the Second Diabetes MILES – Australia Survey; DISTANCE, the Diabetes Study of Northern California; FDS, the Frementle Diabetes Study DIABASIS, French DIABASIS survey LWDS, the Living with Diabetes Study; SLCDC-D, 2011 Survey on Living with Chronic Diseases in Canada – Diabetes component**; **Disease knowledge and information needs – Diabetes mellitus survey**.  ^a^MILES – Australia and MILES-2 are both part of the Diabetes MILES Study International Collaborative, the MILES-2 Survey being partly a follow up study of the 2011 MILES – Australia Survey. Another survey of the Collaborative is the Diabetes MILES – The Netherlands [10] (not included here) which incorporates the same core set of measures as the 2011 MILES – Australia Survey. ^b^Only constructs of the survey component for individuals with diabetes are compared to constructs of the other diabetes surveys. ^c^Variables included in the MILES – Australia. ^d^Variables included in MILES-2. ^e^Assessed in individuals with and without diabetes. ^f^Constructs were not clearly distinguishable. Category subsumes constructs as health-related quality of life, functional health, impact of diabetes on daily life or quality of life. | | | | | | | | | | |

**References**

1. Al Sayah F, Majumdar SR, Soprovich A, Wozniak L, Johnson ST, Qiu W, et al. The Alberta's Caring for Diabetes (ABCD) Study: Rationale, Design and Baseline Characteristics of a Prospective Cohort of Adults with Type 2 Diabetes. Can J Diabetes. 2015;39 Suppl 3:S113-9.

2. Peyrot M, Burns KK, Davies M, Forbes A, Hermanns N, Holt R, et al. Diabetes Attitudes Wishes and Needs 2 (DAWN2): a multinational, multi-stakeholder study of psychosocial issues in diabetes and person-centred diabetes care. Diabetes Res Clin Pract. 2013;99(2):174-84.

3. Speight J, Browne JL, Holmes-Truscott E, Hendrieckx C, Pouwer F. Diabetes MILES-Australia (management and impact for long-term empowerment and success): methods and sample characteristics of a national survey of the psychological aspects of living with type 1 or type 2 diabetes in Australian adults. BMC Public Health. 2012;12(1):120.

4. Browne JL, Holmes-Truscott E, Ventura AD, Hendrieckx C, Pouwer F, Speight J. Cohort profiles of the cross-sectional and prospective participant groups in the second Diabetes MILES—Australia (MILES-2) study. BMJ Open. 2017;7(2).

5. Moffet HH, Adler N, Schillinger D, Ahmed AT, Laraia B, Selby JV, et al. Cohort Profile: The Diabetes Study of Northern California (DISTANCE)--objectives and design of a survey follow-up study of social health disparities in a managed care population. Int J Epidemiol. 2009;38(1):38-47.

6. Davis TM, Bruce DG, Davis WA. Cohort profile: the Fremantle Diabetes Study. Int J Epidemiol. 2013;42(2):412-21.

7. Mosnier-Pudar H, Hochberg G, Eschwege E, Virally ML, Halimi S, Guillausseau PJ, et al. How do patients with type 2 diabetes perceive their disease? Insights from the French DIABASIS survey. Diabetes Metab. 2009;35(3):220-7.

8. Donald M, Dower J, Ware R, Mukandi B, Parekh S, Bain C. Living with diabetes: rationale, study design and baseline characteristics for an Australian prospective cohort study. BMC Public Health. 2012;12(1):8.

9. Baillot A, Pelletier C, Dunbar P, Geiss L, Johnson JA, Leiter LA, et al. Profile of adults with type 2 diabetes and uptake of clinical care best practices: results from the 2011 Survey on Living with Chronic Diseases in Canada - Diabetes component. Diabetes Res Clin Pract. 2014;103(1):11-9.

10. Nefs G, Bot M, Browne JL, Speight J, Pouwer F. Diabetes MILES – The Netherlands: rationale, design and sample characteristics of a national survey examining the psychosocial aspects of living with diabetes in Dutch adults. BMC Public Health. 2012;12(1):925.
