# Supplementary material for: Psychometric properties of a nationwide survey for adults with and without diabetes: the “disease knowledge and information needs – diabetes mellitus (2017)” survey
Source: BMC Public Health. 2020 Feb 6;20:192. doi: 10.1186/s12889-020-8296-6 (PMC7006078; doi:10.1186/s12889-020-8296-6)
Supplement: Supplementary file 2 — Additional file 2. Scale distributions, reliability and unidimensional factor analyses for scales used in both survey components. The table presents scale distributions, Cronbach’s Alpha coefficient as well as results of confirmatory, i.e. unidimensional factor analyses for scales used in the two survey components: people with diagnosed diabetes and people without diagnosed diabetes. [file 12889_2020_8296_MOESM2_ESM.docx]

Additional File 2

| **Table**  *Parameters, reliability and confirmatory factor analyses for scales used in parallel survey components.* | | | | | | | | | | | | |
| --- | --- | --- | --- | --- | --- | --- | --- | --- | --- | --- | --- | --- |
| **Concepts/Constructs** | **Measure or variable** | **People with diabetes (n = 1396)** | | | | | **People without diabetes (n = 2327)** | | | | | |
|  |  | **M** | **SD** | **α^a^** | **RMSEA** | **CFI** | **Measure or variable** | **M** | **SD** | **α^a^** | **RMSEA** | **CFI** |
| **Disease perceptions and believes** | | | | | | | | | | | | |
| Relative risk perception: Optimistic bias | Optimistic bias subscale (2 items, RPS-DM)^b,e^ [1] | 2.6 | 2.7 | .71 |  |  | Optimistic bias subscale (2 items, RPS-DD)^b,f^ [2] | 2.3 | 0.7 | .65 |  |  |
| Perceived personal control | Personal control subscale (4 items, IPQ-R)^b,g^ [3, 4] | 16.0 | 2.8 | .76 | .21 | .91 | Personal control subscale (4 items, RPS-DD)^b,h^ [2] | 1.9 | 0.5 | .43 | .19 | .73 |
| Diabetes-specific self-efficacy | Self-care ability subscale (4 items adapted from the DCP, 1 item modified)^i^ [5] | 3.6 | 0.4 | .53 | .06 | .98 |  |  |  |  |  |  |
| Diabetes-related stigmatization^j^ | 2 Items adopted from the Diabetes Representative Survey [6], 1 new item based on the DSAS-2^c,k^ [7] | 2.0 | 0.8 | .52 |  |  | 2 Items adopted from the Diabetes Representative Survey [6], 1 new item based on the DSAS-2^c,k^ [7] | 2.5 | 0.7 | .31 |  |  |
| **Knowledge about disease** | | | | | | | | | | | | |
| Actual diabetes knowledge |  |  |  |  |  |  | 2 Items adopted from Hoghton et al. [8]; 4 new items^l^ | 2.5 | 1.6 | .62^m^ | .10 ^m,n^ | .94 ^m,n^ |
| **Psychological aspects and health** | | | | | | | | | | | | |
| Diabetes distress | PAID-5 (5 items)^o^ | 3.4 | 4.3 | .88 | .13 | .97 |  |  |  |  |  |  |
| Depressive symptoms | PHQ-2 (2 items)^p^ [9] | 1.2 | 1.6 | .65 |  |  |  |  |  |  |  |  |
| **Health care** | | | | | | | | | | | | |
| Patient-provider-relationship (patient assessed care) | PACIC-DSF (9 items)^q^ [10] | 2.5 | 1.0 | .85 | .07 | .96 |  |  |  |  |  |  |
| **Diabetes-related information** | | | | | | | | | | | | |
| Perceived level of information | IND (11 items)^d,r^ [11] | 2.7 | 0.6 | .90 | .11 | .90 | IND [11] (5 items)^d,r^ | 2.3 | 0.7 | .91 | .07 | .99 |
| *Note*. RMSEA, root mean square error of approximation; CFI, comparative fit index; DCP, Diabetes Care Profile; DSAS-2, Type 2 Diabetes Stigma Assessment Scale; IND, Information Needs in Diabetes Questionnaire; IPQ-R, Revised Illness Perception Questionnaire; PACIC-DSF, Patient Assessment of Chronic Illness Care-DAWN Short Form; PAID-5, Problem Areas in Diabetes Scale – Five-item Short Form ; PHQ-2, Two-item Patient Health Questionnaire; RPS-DD, Risk Perception Survey-Developing Diabetes; RPS-DM, Risk Perception Survey-Diabetes Mellitus.  ^a^For scales comprising two items the standardized coefficient alpha is reported.  ^b^Constructs were similar regarding content but assessed with different measures.  ^c^Items were identical regarding content but phrased from different perspectives (with vs. without diabetes).  ^d^Items were fully identical in both survey components.  Theoretical scale range (higher scores representing higher level of the construct)  ^e^Optimistic bias subscale: 1-4.  ^f^Optimistic bias subscale: 1-4.  ^g^Personal control subscale: 4-20.  ^h^Personal control subscale: 1-4.  ^i^Self-care ability subscale: 1-4.  ^j^N = 1096: sample with diabetes and N = 1502: sample without diabetes. Scales comprised a “don’t know” category to avoid refused responses. Incomplete cases were excluded from analyses and reported separately (Additional File 4, Supplementary Table 1 and Supplementary Table 2),  ^k^Diabetes-related stigmatization: 1-5.  ^l^Actual diabetes knowledge: 0-6. Items could be answered with “true”, “false” or “don’t know”. Missing values due to use of the “don`t know” category or to refused responses were not imputed but counted as non-correct answers and were reported separately (Additional File 4, Supplementary Table 3).  ^m^N = 2318 (Individuals who refused to respond were excluded from the analyses. The answer categories “false” and “don`t know” were combined).  ^n^CFA was based on unweighted data as the R package lavaan.survey did not support conducting CFA on dichotomous items when applying sample weights. For dichotomous items a diagonally weighted least square estimator (WLSMV) was used and the scaled RMSEA and CFI were reported.  ^o^PAID-5: 0-20.  ^p^PHQ-2: 0-6.  ^q^PACIC-DSF: 1-5.  ^r^IND: 1-4. | | | | | | | | | | | | |

**References**

1. Walker EA, Caban A, Schechter CB, Basch CE, Blanco E, DeWitt T, et al. Measuring comparative risk perceptions in an urban minority population: the risk perception survey for diabetes. Diabetes Educ. 2007;33(1):103-10.

2. Walker EA, Mertz CK, Kalten MR, Flynn J. Risk perception for developing diabetes: comparative risk judgments of physicians. Diabetes Care. 2003;26(9):2543-8.

3. Moss-Morris R, Weinman J, Petrie KJ, Horne R, Cameron LD, Buick D. The revised Illness Perception Questionnaire (IPQ-R). Psychology & Health. 2002;17(1):1-16.

4. Gaab J, Bunschoten SL, Sprott H, Ehlert U. Psychometric evaluation of a German translation of the Illness Perception

Questionnaire. 2004.

5. Fitzgerald JT, Davis WK, Connell CM, Hess GE, Funnell MM, Hiss RG. Development and validation of the Diabetes Care Profile. Eval Health Prof. 1996;19(2):208-30.

6. Ratgeber D. Diabetes. Repräsentativ-Befragung. Ein Studie der GfK Marktforschung im Auftrag des Diabetes Ratgebers. Diabetes Ratgeber. Baierbrunn: Wort und Bild Verlag; 2016.

7. Browne JL, Ventura AD, Mosely K, Speight J. Measuring the Stigma Surrounding Type 2 Diabetes: Development and Validation of the Type 2 Diabetes Stigma Assessment Scale (DSAS-2). Diabetes Care. 2016;39(12):2141-8.

8. Hoghton MR, Philipp R, Harvey K, Hughes AO, Fletcher GM, Burns Cox CJ. Understanding of diabetes mellitus in non-diabetic adults. Practical Diabetes. 1987;4(2).

9. Kroenke K, Spitzer RL, Williams JB. The Patient Health Questionnaire-2: validity of a two-item depression screener. Med Care. 2003;41(11):1284-92.

10. Nicolucci A, Kovacs Burns K, Holt RIG, Comaschi M, Hermanns N, Ishii H, et al. Diabetes Attitudes, Wishes and Needs second study (DAWN2™): Cross-national benchmarking of diabetes-related psychosocial outcomes for people with diabetes. Diabetic Medicine. 2013;30(7):767-77.

11. Chernyak N, Stephan A, Bachle C, Genz J, Julich F, Icks A. Assessment of information needs in diabetes: Development and evaluation of a questionnaire. Prim Care Diabetes. 2016;10(4):287-92.
