# Supplementary material for: Psychometric properties of a nationwide survey for adults with and without diabetes: the “disease knowledge and information needs – diabetes mellitus (2017)” survey
Source: BMC Public Health. 2020 Feb 6;20:192. doi: 10.1186/s12889-020-8296-6 (PMC7006078; doi:10.1186/s12889-020-8296-6)
Supplement: Supplementary file 3 — Additional file 3: Table S1, S2 and S3. For questions on disease-related stigmatization in both people with and without known diabetes and actual diabetes knowledge in people without known diabetes, numbers of participants that selected “don’t know” as a response or refused to answer as well as corresponding sample characterization are presented in Table S1, S2 and S3. [file 12889_2020_8296_MOESM3_ESM.docx]

**Additional file 3**

For questions on disease-related stigmatization in both people with and without known diabetes and actual diabetes knowledge in people without known diabetes, numbers of participants that selected “don’t know” as a response or refused to answer are presented in Table 1, Table 2 and Table 3.

| **Supplementary Table 1.**  *Diabetes-related stigmatization scale in people with diabetes: Sample characterization by response pattern.* | | | | |
| --- | --- | --- | --- | --- |
|  | Number of items answered with “Don’t know” or refused to answer | | | |
|  | No item (n = 1096)^a^ | One item (n = 240)^a^ | Two items (n = 53)^a^ | Three items (n = 7)^a^ |
| Age in years, mean (SD) | 64.9 (13.7) | 65.9 (14.4) | 68.2 (11.9) | 73.8 (13.1) |
| Sex (% female), % | 49.8 | 52.0 | 38.9 | 58.3 |
| Educational level^b^, %  Low  Average/medium  High | 45.3  40.8  13.9 | 50.7  35.7  13.1^c^ | 57.4  32.5  10.0 | 55.9  37.7  6.4 |
| *Note*. Total N_unweighted_ = 1396. Data are given as weighted. Stigmatization was assessed with three items using a 5-point Likert scale response format from ‘completely disagree’ to ‘completely agree’ with an additional response option ‘don’t know’.  ^a^Sample sizes are reported unweighted.  ^b^Comparative Analysis of Social Mobility in Industrial Nations (CASMIN) classification [1] was used to report educational level.  ^c^Information on educational level was missing for n = 2. | | | | |

| **Supplementary Table 2.**  *Diabetes-related stigmatization scale in people without known diabetes: Sample characterization by response pattern.* | | | | |  |
| --- | --- | --- | --- | --- | --- |
|  | Number of items answered with “Don’t know” or refused to answer | | | |  |
|  | No item (n = 1502)^a^ | One item (n = 551)^a^ | Two items (n = 201)^a^ | Three items (n = 73)^a^ | |
| Age in years, mean (SD) | 48.1 (18.0) | 52.3 (18.6) | 54.4 (19.3) | 49.7 (21.2) | |
| Sex (% female), % | 51.9 | 51.7 | 51.7 | 48.4 | |
| Educational level^b^, %  Low  Average/medium  High | 26.7  44.8  28.4^c^ | 31.5  41.4  26.9^c^ | 39.5  36.1  24.0^c^ | 65.7  21.2  12.8^c^ | |
| *Note*. Total N_unweighted_ = 2327. Data are given as weighted. Stigmatization was assessed with three items. Each item used a 5-point Likert scale response format from ‘completely disagree’ to ‘completely agree’ with an additional response option ‘don’t know’.  ^a^Sample sizes are reported unweighted.  ^b^Comparative Analysis of Social Mobility in Industrial Nations (CASMIN) classification [1] was used to report educational level.  ^c^Information on educational level was missing for n = 1. | | | | |  |

| **Supplementary Table 3.**  *Actual diabetes knowledge in people without known diabetes: Sample characterization by response pattern.* | | | | |
| --- | --- | --- | --- | --- |
|  | Number of items answered with “Don`t know” or refused to answer | | | |
|  | No item (n = 356)^a^ | 1-2 items (n = 904)^a^ | 3-4 items (n = 690)^a^ | 5-6 items (n = 377)^a^ |
| Age in years, mean (SD) | 46.1 (16.6) | 48.3 (17.6) | 51.5 (19.5) | 51.2 (19.3) |
| Sex (% female), % | 61.6 | 56.2 | 47.2 | 44.0 |
| Educational level^b^, %  Low  Average/medium  High | 13.7  41.2  45.0 | 24.0  47.5  28.1^c^ | 38.5  38.5  23.0 | 41.4  38.7  19.6^c^ |
| *Note*. Total N_unweighted_ = 2327. Actual diabetes knowledge in people without known diabetes was assessed with six items, which could be answered with the response categories ‘True’, ‘False’ or ‘Don’t know’.  ^a^Sample sizes are reported unweighted.  ^b^Comparative Analysis of Social Mobility in Industrial Nations (CASMIN) classification [1] was used to report educational level.  ^c^Information on educational level was missing for n = 2. | | | | |

**References**

1. Brauns H, Scherer S, Steinmann S. The CASMIN Educational Classification in International Comparative Research. In: Hoffmeyer-Zlotnik JHP, Wolf C, editors. Advances in Cross-National Comparison. Boston, MA: Springer US; 2003. p. 221-44.
