# Supplementary material for: Psychometric properties of a nationwide survey for adults with and without diabetes: the “disease knowledge and information needs – diabetes mellitus (2017)” survey
Source: BMC Public Health. 2020 Feb 6;20:192. doi: 10.1186/s12889-020-8296-6 (PMC7006078; doi:10.1186/s12889-020-8296-6)
Supplement: Supplementary file 4 — Additional file 4: Table S1 and S2. Exemplary post-hoc structural model modifications were applied to actual diabetes knowledge and perceived personal control which in people without known diabetes. [file 12889_2020_8296_MOESM4_ESM.docx]

**Additional file 4**

Post-hoc structural model modifications were applied to actual diabetes knowledge which was assessed with six items and perceived personal control which was assessed with four items in people without known diabetes.

| **Table 1.**  *Confirmatory factor analysis on actual diabetes knowledge in people without diabetes: model comparison.* | | | | | | | | |
| --- | --- | --- | --- | --- | --- | --- | --- | --- |
| CFA Model | *χ2* | *df* | RMSEA | CFI | *Δχ2*^a^ | *Δdf* | AIC^b^ | BIC^b^ |
| One-factor model with six indicators | 153.10 | 9 | .10 | .94 |  |  |  |  |
| Three-factor model with two indicators on each factor | 16.42 | 6 | .04 | .99 | 146.06*** | 3 |  |  |
| *Note.* N=2318 (individuals who refused to respond were excluded from analyzes). RMSEA, root mean square error of approximation, lower values indicate better fit; CFI, comparative fit index, higher values indicate better fit; Akaike information criterion/Bayesian information criterion, lower values indicate better fit. ***p < .001.  ^a^Model comparison is based on the method ‘satorra.2000’, using a scaled shiftet test statistic.  ^b^AIC and BIC are not provided when WLSMV is used as estimator. | | | | | | | | |

| **Table 2.**  *Confirmatory factor analyses on perceived personal control in people without diabetes: model comparison.* | | | | | | | | |
| --- | --- | --- | --- | --- | --- | --- | --- | --- |
| CFA Model | *χ2* | *df* | RMSEA | CFI | *Δχ2* | *Δdf* | AIC | BIC |
| One-factor model with four indicators | 57.47 | 2 | .19 | .73 |  | 0 | 20732.33 | 20801.35 |
| Two-factor model with two indicators on each factor^a^ | 0.96 | 2 | .00 | 1.00 |  |  | 20553.71 | 20622.73 |
| *Note*. N = 2327. RMSEA, root mean square error of approximation, lower values indicate better fit; CFI, comparative fit index, higher values indicate better fit; AIC/BIC, Akaike information criterion/Bayesian information criterion, lower values indicate better fit.  ^a^Model included a constrained error variance of one item. | | | | | | | | |
